# Modification of kitchen blenders into controllable laboratory mixers for mechanochemical synthesis of atomically thin materials

**DOI:** 10.1016/j.ohx.2023.e00471

**Published:** 2023-09-11

**Authors:** Diego T. Pérez-Álvarez, Jacob Brown, Jason Stafford

**Affiliations:** School of Engineering, The University of Birmingham, Edgbaston, Birmingham, B15 2TT, United Kingdom

**Keywords:** 2D materials, Graphene, Liquid phase exfoliation, Shear mixing, Scalable

## Abstract

Graphene and related two-dimensional materials (2DMs) have shown promise across numerous technology areas including flexible electronics, energy storage and pollution remediation. Research into novel applications of these atomically thin materials relies on access to synthesis techniques for producing 2DMs with suitable quality and quantity. Liquid-phase exfoliation is a mechanochemical approach that can achieve this and produce defect-free nanomaterial dispersions which are compatible for downstream use (e.g. inkjet printing, coatings). Here, using kitchen blenders to deliver shear-driven exfoliation, we develop a range of inexpensive hardware solutions that can allow researchers to synthesise 2DMs using a controllable, sustainable and scalable process. Extensive modifications were necessary as the onboard electronics lack the experimental controls (temperature, speed, characterisation) for scientific research and precision synthesis. The technical aspects (including the many lessons learned) of the modifications are discussed and a simple selection process is proposed for creating bespoke mechanochemical processors for any application in the hope that this encourages experimentation. Specific builds with detailed notes, cost breakdown and associated files are provided in the Open Science Framework (OSF) repository, OpenLPE associated with this article.

## Graphical abstract

**Figure:**
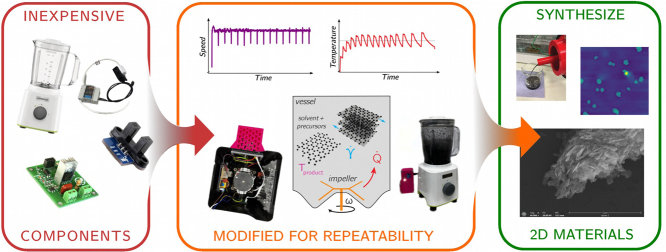

**Table:** 

Specifications table	

Hardware name	OpenLPE

Subject area	• Engineering and material science • General

Hardware type	• *Material synthesis using liquid exfoliation*

Closest commercial analog	Waring LB20ES (£1159). *Though this does not have the full feature set our equipment has.*

Open source license	GPL v3.0

Cost of hardware	£100→£200

Source file repository	https://doi.org/10.17605/OSF.IO/EVUPY

## 1 Hardware in context

Owing to their remarkable optical, mechanical and electrical properties over bulk counterparts [1], nanosheets have shown immense promise in numerous applications from energy storage and generation to wearable healthcare technologies [2]. Although these exciting materials have the potential to transform future technologies, they are challenging to synthesise using sustainable routes that avoid harsh chemicals and toxic solvents. It is also expensive to purchase research-grade materials for scientific investigations (1 g of “Graphene” currently retails at £800 on Sigma Aldrich). To compound this issue, graphene is the most commercially mature Two-Dimensional Material (2DM). The majority of the many dozens of other 2DMs (e.g., semi-conductors and insulators) are not as easy to source. Off-the-shelf materials also require a complete characterisation to confirm their quality is acceptable, primarily due to the prevalence of poor quality products that have been found in the market [3]. This added cost and uncertainty for purchased nanomaterials can price out many researchers, stifling innovation, and negatively impact the translation of ideas into real-world technologies.

One of the most democratising steps in nanosheet production in the past decade has been the use of standard household kitchen blenders to effectively produce a variety of different 2DMs such as Graphene, MoS_2_, h-BN, WS_2_ and others [4], [5], [6], [7] through the mechanism of Liquid Phase Exfoliation (LPE) [8]. The low-cost and widespread availability make this equipment attractive for laboratory investigations. Importantly, impeller-driven mixing and blending processes have been scaled-up for many industrial chemical engineering applications over several decades. Therefore, this simple shear-exfoliation system can support translation pathways for laboratory research, facilitating widespread industrial uses for 2DMs compared to today’s more common laboratory synthesis methods that are inefficient at large-scales (e.g., ultrasonication). A significant limitation, however, is that kitchen blenders are not designed for nanomaterial synthesis and lack the necessary experimental controls for scientific research. The quality of a 2DM has a significant impact on its material properties, emphasising the importance of precise synthesis control and characterisation when it comes to research. Modifications are mandatory due to this, and by the fact that increasing the level of automation increases the reliability and repeatability of the synthesis process.^1^ This work describes how to accommodate this and maintain a low-cost for this scientific instrument. At a minimum, the most modest modifications begin at controlling the power cycling of the blender (see Section 4.1) and end at the precise control over the speed of the blender (Section 4.4) and process temperature (Section 4.5). Similar commercial blenders are open-loop and do not support power cycling as a rule.

**Fig. 1:**
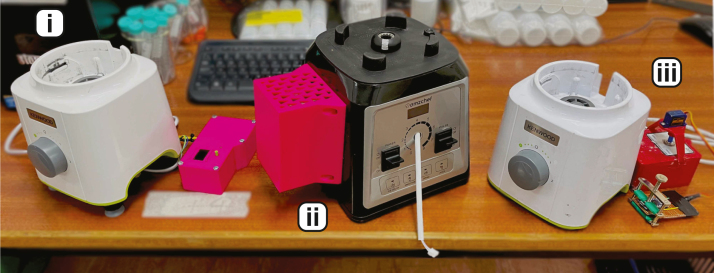
Some examples of modified blenders tested in our lab. (i) is a Kenwood modified in accordance with Sections 4.2, 4.3, 4.4 (with option B from Table 2) and includes a screen for a digital readout. (ii) is an AMZchef with variable speed (Section 4.2, uses option C from Table 2) and is typically used with the programmable extension lead in Section 4.1. (iii) is a modified Kenwood with option D from Table 2, and has an added servo to facilitate the *on*/*off* cycling. Software and build guides can be found on our Open Science Framework (OSF) page.

Nowadays, there are numerous examples of open-source equipment which use some form of 2DMs in solution (such as conductive inks) to create functional devices [9], [10], [11]; but simultaneously, there are few such open-source projects for the production of 2DMs. The only recent example the authors could find of an open-source framework for scalable production of 2DMs is AutoCVD [12]. This bottom-up approach uses Chemical Vapour Deposition (CVD) to grow 2D materials on copper substrates. Although this method produces large-area 2DM thin-film deposits which can be useful in sensing and electronics, it is not suitable for producing large quantities of 2DM as required for functional inks and composites (typically ∼1–100 g/L). LPE is a top-down approach that has been proven using sonicating probes [13], ultrasonic baths [14], jets [15], industrial homogenisers [16] and more [17], though on the whole, these systems are either prohibitively expensive or offer little advantage to the standard kitchen blender when comparing 2DM concentrations and production rates. Table 1 juxtaposes some modified blenders from this work (see Fig. 1) to the nearest commercial equivalent lab blender (Waring LB20ES). Though the commercial blender has greater chemical resistance due to its use of a stainless steel vessel, the modified alternatives are both cheaper and of similar or higher power.

**Table 1:** A comparison of current commercial blender offerings and some of this works’ modified alternatives.

The hardware in this paper is modular, hence we leave it to the reader to select from the range of modifications what suits their research best. All setup information can be found in more detail on the Open Science Framework (OSF) page OpenLPE. Furthermore, we give insights into the preprocessing, analysis, characterisation and process intensification which will aid researchers in manufacturing their own 2DMs reliably in-house.

## 2 Hardware description

In its simplest form, the equipment is a standard kitchen blender modified to conduct an *on*/*off* cycling procedure to ensure the batch and high-power internal motor do not overheat. The choice of blender is left to the readers’ discretion, however all blenders we have examined utilise similar design AC motors, making the power electronics straightforward to regulate. The cost of the base blender tends to parallel the increasing complexity in power regulation, power output, and the chemical resistance of the vessel. Synthesis occurs by exerting mechanical force on the precursor/solvent mixture, which leads to particle break-up and produces shear rates that are dependent on the rotational speed of the impeller ($\overset{˙}{\gamma }∼\omega^{3/2}$ [18]). Adding speed control ensures the conditions within the blender are precisely set, allowing for more selective and repeatable synthesis.

The ease of use of this type of system, coupled with the synthesis information and characterisation of production given here, allows anyone to reliably control the synthesis of 2DMs for their own research application. The only other systems that come close in terms of simplicity include the original scotch-tape method [19], and the modified Hummer’s method [20]. LPE offers a few distinct advantages here:

• One can choose to use green solvents (depending on vessel compatibility), thereby making the process non-toxic and environmentally friendly.
• It is a straightforward approach that requires minimal set up.
• 2DMs manufactured using LPE maintain good quality as the process does not introduce additional nanosheet defects (e.g., oxidation, basal plane defects).
• It is the most cost effective system currently available. We estimate that the equivalent of 1 g of Few Layer Graphene (FLG) can be produced (average number of atomic layers 〈N〉<10) in a water-surfactant solution in one hour of processing for less than £200 (including capital costs).

Graphene and related materials have already been shown to have an enormous impact within many application areas [2]; hence, we hope that by sharing the build process and documentation, as well as pitfalls and lessons learned, we can increase accessibility and innovation around these novel nanomaterials.

## 3 Designs summary

*All files are accessible directly from the OSF page*OpenLPE.

**Table:** 

Design	Cost	Open source license	Location of the Repository
PID Mixer	£100	GPL v3.0	DTP587/PID-Blender

Triac Mixer	v1: £100	GPL v3.0	DTP587/Triac-Blender
	v2: £170	GPL v3.0	DTP587/Triac-Blender-v2

Microcontroller Relay	£65	GPL v3.0	stafforj/Automated-Relay-Switch

Inline material characterisation	…	CC BY 4.0	[18]

Cooling system	Datalogger: £165	GPL v3.0	stafforj/Thermocouple-Datalogger
	Cooling unit: £105	GPL v3.0	stafforj/Low-Cost-LPE-Cooling-System

*For every design file in the summary table above, The authors have included an in depth description of the functioning in* Section 4*. Relevant Bill Of Materials (BOM) are stored within their respective repositories.*

## 4 Build instructions

The systems described in this article are all inherently modular, with the blenders themselves being entirely interchangeable with any other commercial blender due to the standardised use of mains supply AC motors. Each sub-system is described individually, giving a brief overview of their function and application, including build notes. More details about these sub-systems and how they are applied can be found on our OSF page. Components required for each modification are listed in the BOM.

### 4.1 On/off cycling

*On/off cycling or switching is essential to avoid overheating and maintain device reliability.*

Commercial blenders have restrictions in their duration of continuous operation. This is to prevent overheating of the motor and thermally-induced failures of the mixing vessel components. To run a material synthesis for the typical process times used in LPE (e.g., ∼10–100 mins), an automated method to perform *on*/*off* cycling without user intervention significantly reduces manual efforts. This requires only three adjustments to the Arduino code “relay-switch.ino” before beginning a material synthesis. The process *on* time interval (ms) is set by adjusting  ; process *off* time interval (ms) is set by adjusting  ; and the total process *on* time (ms) required of the synthesis operation is set by adjusting the maximum time to be  . In this example, the blender operates for 1 min *on*, 2 mins *off* for a total *on* time of 30 mins. After the total *on* time is reached, the relay reverts to a normally-open condition and the blender remains *off*.

**Fig. 2:**
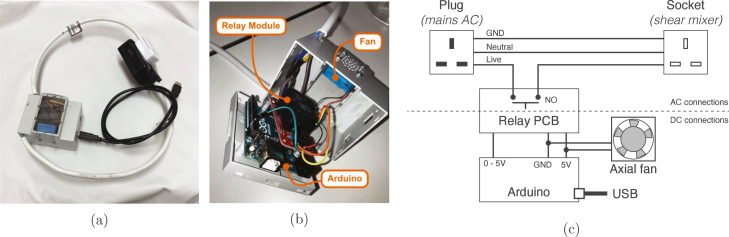
Arduino-controlled *on*/*off* extension lead. This automated relay switch controlled many of the appliances listed in this work, and can be combined with other sensors for dynamic, closed-loop control.

Note that for certain blenders, such as the AMZChef in Table 1, *on*/*off* cycling as described here is difficult due to interference from the onboard electronics. If this is the case, consider the installation with a triac module (see Section 4.4). Simpler blenders such as this Kenwood model have no in-built software to detect shut-off, so this basic addition is typically all one needs. To avoid failures during experiments, we found it was essential to follow the manufacturer’s recommendations and alternate running 1 min *on* together with an *off* time that ensures sufficient cooling of the product batch and internal motor. To avoid tampering with any on-board electronics, this modification is made completely separate to the blender, as a programmable extension lead (see Fig. 2).

### 4.2 Phase angle control

*Phase angle control allows for accurate power delivery to the blender rotor. This in turn allows for a more exact operating condition, and is a necessary step depending on the blender chosen. Phase angle control modules with microcontroller interfaces supersede* Section 4.1*, as on–off cycling can be directly implemented within the arduino code.*

Phase angle control in brief is a way of regulating the duty cycle of an AC signal. Dimming in this context is inherited from the primary use of these circuits as dimmer switches. The function of these triac dimming circuits^2^ (Triode for Alternating Current) is simple in principle, as they act as a diode in the direction of a voltage applied to the gate and simply latch open until the voltage inverts, where it reverts to closed. This simple technology is available as a standalone box with a potentiometer (e.g., [21]) or with an isolated zero-cross interface (see Table 2) suitable for use with an arduino or similar microcontroller (Section 4.4). The wiring is identical for both cases:

**Table 2:** A comparison of current commercial triac modules from several sources, their prices, and a brief note on their suitability in general.

**Fig. 3:**
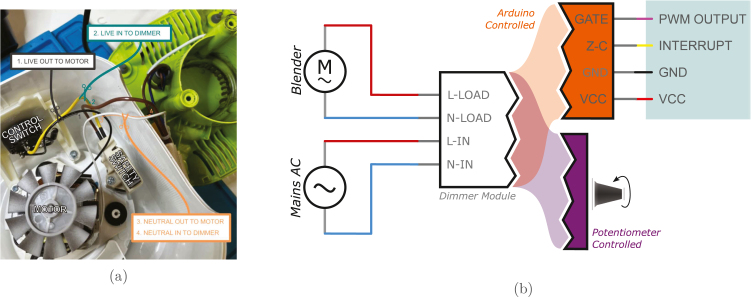
Visual guide to wiring a dimmer module (either arduino or potentiometer control) into a commercial blender, in this case the Kenwood BLP31.D0WG. The safety switch is left inline with the motor in (A), to add an extra layer of security in the unlikely event the vessel detaches from the blender. (B) shows the wiring diagram for either arduino controlled or potentiometer controlled dimming modules.

As blenders are mostly powered by brushed AC motors, the wiring of the triac module is directly in series with the motor, regulating the live signal from mains voltage (see Fig. 3). Hence wiring is simply a matter of snipping two wires and wiring the input of the blender to the output of the triac module, then plugging it into AC. It goes without saying that **AC power is deadly, and though this seems simple, please ensure that you are aware of the risks and how to minimise them before proceeding**. Ensure you select a triac circuit with a similar power rating as the blender you are using, but note that the power dissipated through the triac is typically quite low.

Zero-cross detection is the typical interface for most of the commercially available phase angle control circuits, and the majority consist of four pins; three inputs (power, gate and ground) and one output (zero-cross). To give a small overview of how these boards function, the zero-cross signal pulses high when the AC voltage into the board changes potential (through an in-built zero-cross detection circuit), while the gate input controls a switch which determines whether the signal to the gate pin of the triac is on or off. These circuits are often opto-isolated, but even with this isolation, Electro Magnetic Interference (EMI) can still be an issue and some shielding may be required, especially if the control board is close to the phase angle control circuitry. The circuitry can be controlled using an Arduino compatible board. Begin by attaching the zero-cross pin of the module to an interrupt on the board (  ) and the gate pin to a PWM output (  ):

**Figure:**
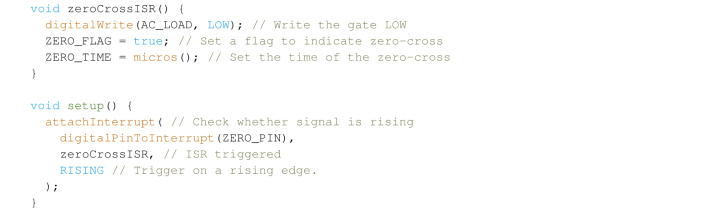

This sequence effectively sets a flag and a timer when the nominal 50 Hz, 230 VAC signal from the mains crosses zero, irrespective of sign. As one period at 50 Hz is approximately 20 ms, a half period crosses zero at every 10 ms. With a suitable delay, setting the gate high after a delay between 0 *on* and 10,000μs *off* effectively controls the duty cycle of the AC signal. Note that this leaves a limited number of operations between zero-crosses, therefore process intensive functions between loops must be kept to a minimum in order to signal the gate in time. Continuing this example, one can simply set a time which correlates to the set speed (  ):  ) desired. This can be calibrated with an optical tachometer, but note that this correlation is actually only matching an inputted power to a  , varying the impeller loading will break the relationship between the two (e.g., due to a change in fluid viscosity).

**Figure:**
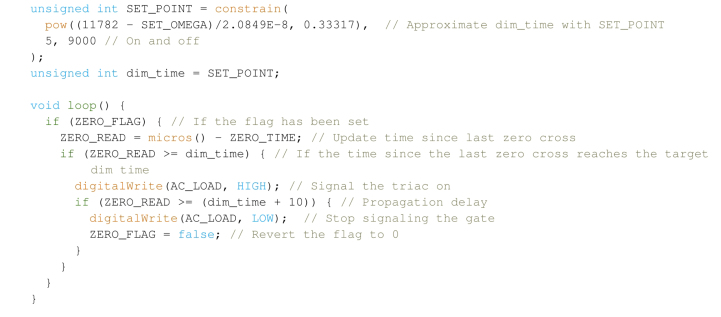

The above loop checks if the flag has been set by the Interrupt Service Routine (ISR)  , which if true establishes the time since the last trigger; if this time is larger or equal to the phase angle (or dimming time), the loop then waits until a short propagation delay (10μs for most triacs) has been reached, at which point the flag is reverted and the gate pin is set back to  . Though this example could be handled in a single ISR, it leaves little scope for modifications to the code. This method provides good stability in the rotor speed, and at most can vary between ±1,000 RPM. It especially fails under different loading conditions, i.e. different vessels and liquids, requiring measurements in order to set the speed accurately every time. Some board manufacturers have released Arduino header files to streamline the integration of these boards into your own projects, but we found our simple implementation to work well without this.

### 4.3 Speed sensor

*Adding a speed sensor facilitates precise impeller speed measurements.*

**Fig. 4:**
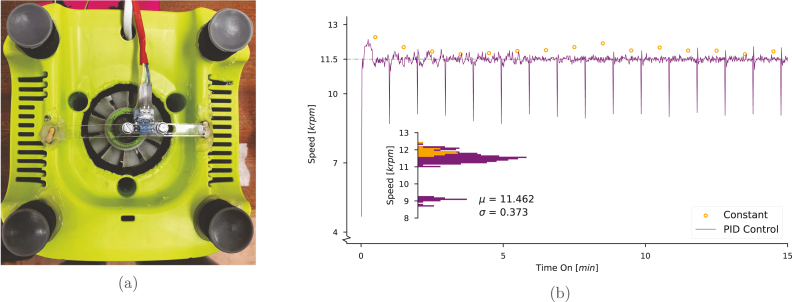
Sensor modifications in (A), the photo-interrupt module was brought up and away from the fan, with a small 3D printed interrupting ring fit into the fan itself. (B) shows the speed of the first 15 min *on* of a run, with a constant  (in orange, measured with an optical tachometer), and a Proportional Integral Differential (PID) control circuit (in purple, measured with an on-board sensor). Inset in (B) is a histogram of speeds (**log scale**). For the “Constant” (orange) case, the speed was carefully measured as stable with the tachometer before the equipment was power cycled and the measurements taken. Note the much lower sampling rate (1 per minute) and the steady state error between the constant and PID cases. (For interpretation of the references to colour in this figure legend, the reader is referred to the web version of this article.)

Potentiometer-controlled dimmers (option D, Table 2) can accurately maintain the speed to a reasonable range (± 1000 rpm between experiments), but there can be significant variability especially at low speeds. Fig. 4(b) shows a snapshot of the first 15 min of a synthesis run, and we can see that after having found a set point for 11.5 krpm with a tachometer, a constant  shows a steady state error from the set point. More expensive blenders such as the AMZchef (in Table 1) have in-built zero-cross and speed sensing circuitry, making it useful for these requirements. Lower cost models, such as the Kenwood described here, do not have an integrated speed sensor and require modification. Modifying the Kenwood involved removing part of the housing to get access to the cooling fan at the bottom of the machine to then attach an interrupting ring,^3^ for a precise reading directly from the motor shaft. It also involved printing extensions for the blenders’ rubber feet, and a bar to mount the photo-interrupter across the gap in the housing. These modifications can be seen in Fig. 4(a).

Speed measurements can be calculated as a counts per time or time per counts basis. Both have their own advantages; time per counts is accurate at low speeds and can fail at higher speeds where the time between counts is very low, counts per time has a set resolution depending on the number of sections in the photo-interrupting strip. In this case, we chose to go with the simpler counts per time basis, which with 1 s between readings and single gap in the interrupting strip gives a resolution of 60 rpm, as this was found to be a good balance of accuracy at both extremes. This is implemented as an ISR in the arduino code:

**Figure:**
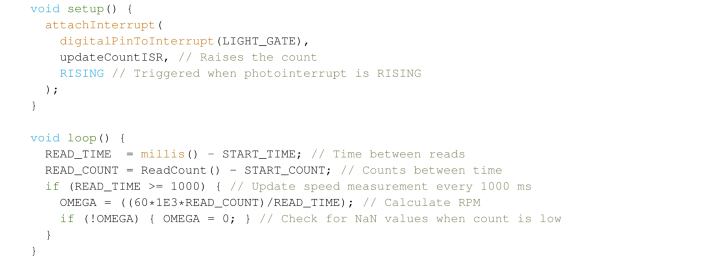

The above stores and updates the blender speed every second as the variable  . This can be used by itself as a speed sensor without any further modification, though at this point it becomes trivial to implement closed loop control if one has already made the modifications in Section 4.2.

### 4.4 PID control

*Speed sensing* 4.3 *coupled with phase angle control* 4.2 *allows for closed-loop speed control, to maintain a precise speed.*

Though the AMZchef has many of the features we need natively; unfortunately, speed is controlled through a basic relay-switching type circuit, where power is regulated by simply switching relays on or off as quick as they will allow. This method again works to regulate the duty cycle of the AC signal, but instead of regulating every half wave, the cycle is every tens or twenties of cycles, which does not allow for the precise speed control required for scientific research. For this reason, we decided on closed loop control by integrating a speed sensor into the blender. This is achieved through implementation of a PID controller, as it is simple to implement and easy to tune. By comparing a target speed to the actual speed every time  is calculated, one returns an error term, which can then feedback into our value for  .

**Figure:**
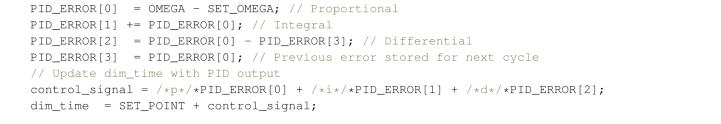

This short section of code calculates the different PID terms and then goes on to modify the  accordingly. In Fig. 4(b), shows the rotational speed of the blender over the first 15 min *on* of a run (Manufacturer recommendation is to allow for 1 min *off* after every minute *on* to avoid overheating), and shows that the speed is maintained very accurately except during the first second of the run when the rotor is still in the process of attaining its target speed. After the first cycle, the PID controller is set not to update after the first reading, so that this initial rise in velocity does not perturb the set point discovered in previous cycles. The inset of Fig. 4(b) is the speed represented as a histogram, where the variation from the set point is shown.

### 4.5 Cooling jacket

*Adding a cooling jacket around the vessel can help control the temperature of the batch during synthesis and ensure the blender components maintain reliable operating temperatures.*

**Fig. 5:**
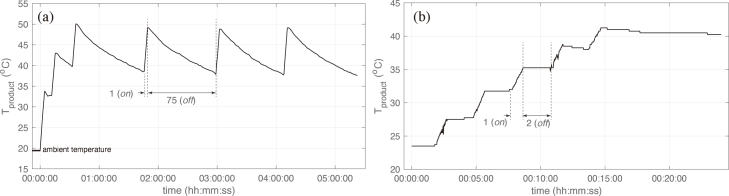
Batch temperature during the synthesis of few-layer graphene in water-sodium cholate using (a) “bullet“ style and (b) “jug” style process vessels. All other synthesis parameters were kept the same across both cases including $C_{i}=20$ g/L (graphite concentration), $C_{sc}=4.3$ g/L (sodium cholate concentration), V=200 mL (product volume) and ω=20,000 rpm (impeller speed).

**Fig. 6:**
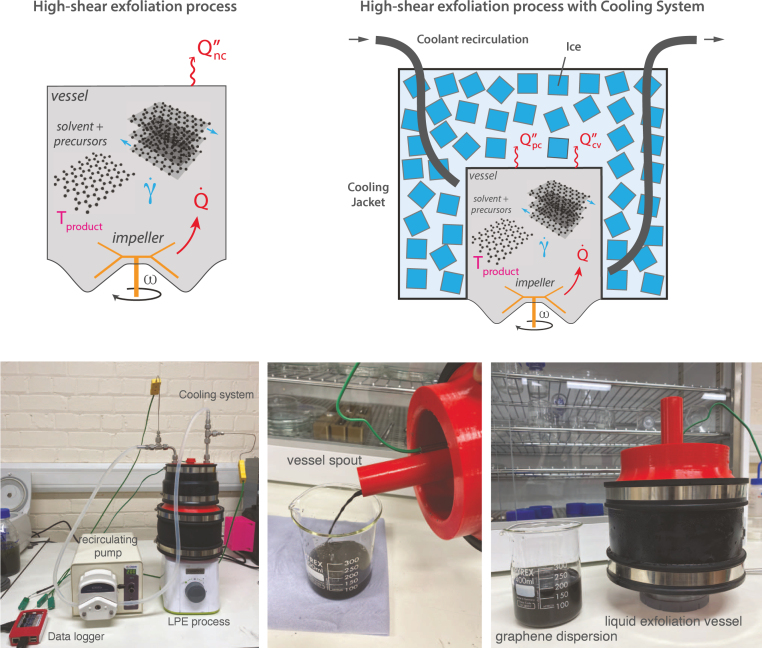
The cooling system utilises the phase change of ice (latent heat of fusion) to remove heat ($Q_{pc}^{\prime \prime }$) from the Liquid Phase Exfoliation (LPE) process. The cooling jacket is constructed using 3D-printed polyethylene terephthalate glycol-modified (PETG) parts and two large ethylene propylene diene monomer (EPDM) pipe connectors. A thermocouple board is connected to a Raspberry Pi 3B+ to record and plot temperatures from type K thermocouples in real-time. The addition of precursors and product sampling is possible using the vessel spout. When the synthesis process is completed, the product is emptied from this vessel spout also.

Higher product yields can be achieved by exposing the precursor and solvent mixture to higher strain rates more frequently or for longer residence times [18], [22]. For the high shear blender, this can be achieved by reducing the vessel size. Active temperature control becomes a requirement when moving from large volume “jug” vessels (≈1–2L) to smaller volume “bullet” vessels (≈0.1–1L, see Fig. 9). It may also be necessary for controlling mechanochemical synthesis when chemical reactions that are sensitive to temperature are present. Heat is generated in the LPE process through viscous heat generation as the fluid experiences turbulent shear stresses. This is illustrated in the schematic in Fig. 6, where $\overset{˙}{Q}$ is the volumetric heat generation and $Q_{nc}^{\prime \prime }$ is the natural cooling to the environment. The practical issue when moving to small vessel geometries is the reduction in heat transfer to the ambient surroundings. The batch temperature ($T_{product}$) can increase to/above the point where there is a risk of thermomechanical failures to the vessel and/or rotor coupling between the impeller and motor. Temperature rises can occur rapidly (≈12°C/min), as shown in Fig. 5 which plots the batch temperature during the exfoliation of graphite into FLG using the two different styles of vessels. After just three minutes (*on* time), the temperature increases by 36°C when operating with a “bullet” style vessel. The corresponding increase when using the “jug” style vessel was only 12°C. One way to address this is to set the *off* time to a sufficiently long duration, however, the thermal capacitance of these systems can require an excessive *off* time that can be impractical. Indeed, we found that *off* time intervals of up to 75 mins were necessary to avoid the batch exceeding 50°C when leaving the system to cool naturally in ambient air (see Fig. 5 a). In contrast, the synthesis of FLG using the “jug” vessel required *off* time intervals typically of 1–3 mins (Fig. 5 b).

An alternative approach that reduces the *off* time interval and maintains process temperature control is to introduce active cooling. This build involved two main components: (i) a cooling jacket that efficiently transfers heat from the process and (ii) temperature acquisition to enable process monitoring and control. To avoid the requirement for expensive chillers that may not be accessible to every lab, we designed the cooling system to function using ice cubes/chilled water as a coolant (note the system can also be operated with a chiller if this is available). Illustrations of the cooling assembly and its breakdown, including the type K thermocouple data logger are shown in Fig. 6. Detailed build instructions, CAD files and programming codes are available in the two repositories provided in Section 3.

**Fig. 7:**
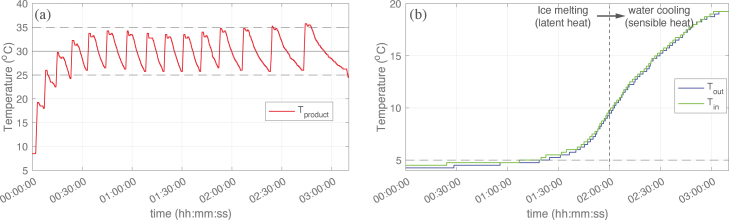
The temperatures of the (a) batch and (b) coolant during the synthesis of few-layer graphene in water-sodium cholate using a “bullet” style process vessel with integrated cooling system. The synthesis parameters were kept the same as for the cases without cooling in Fig. 5 including $C_{i}=20$ g/L, $C_{sc}=4.3$ g/L, V=200 mL and ω=20,000 rpm.

This system can cool the LPE process to sub-ambient temperatures and/or maintain batch temperature below a target limit. To demonstrate this, Fig. 7 presents the batch temperature during a 15 min synthesis of FLG in an aqueous-surfactant solution where the *off* time was dynamically adjusted to target an average product temperature of 30±5°C. The inlet and outlet temperatures of the coolant demonstrate the change in cooling modes (latent and sensible heat). For 1.5 h (10 mins *on*), the coolant temperature increases by ≈2°C and much of the heat generated by the shear exfoliation process goes into melting the ice cubes. Then, as most of the ice melts, the coolant temperature increases >15°C and the heat is removed by convection. In this operating region, longer *off* times are necessary to maintain the target product temperature. It is recommended that users size their cooling system and ice packing levels to meet their required process times and impeller power input. A basic thermal analysis is provided in the design files that the reader can use as a guideline for their LPE process (Section 3).

### 4.6 In-line monitoring

*In-line monitoring can be used to assess 2DM production in parallel to other *ex situ* characterisation methods.*

**Fig. 8:**
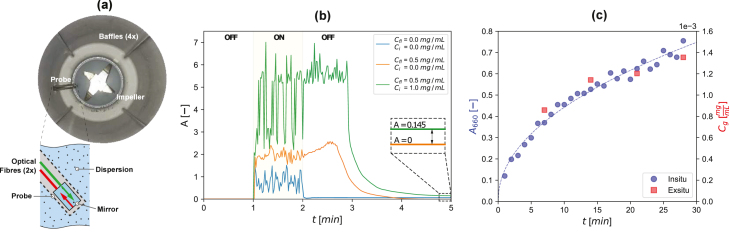
The fibre-optic probe mounted in a position above the impeller in part (a) with a small inset schematic detailing its function. (b) compares manual measurements taken exsitu to measurements taken with the probe for a similar 15 min run. (c) shows the optical noise generated in between the probe and the mirror. Reproduced with permission [18].

A significant challenge from lab to industrial scales of graphene and other 2DM production is the lack of high-throughput materials characterisation tools. Rapid optimisation of LPE processes and batch-to-batch material quality assurance is restricted as a consequence. To address this, our group conducted in-line monitoring of few-layer graphene production within these blenders using an optical spectroscopy method that has been documented in previous work [18]. This method integrates UV–vis–NIR spectroscopy by suspending a fibre-optic probe within the liquid being agitated in the mixer (see Fig. 8). To conduct our measurements, a hole was cut into the side of the vessel and a probe holder was 3D-printed and bonded to the side wall. This allowed the fibre-optic probe to be placed inside the vessel just above the impeller blades. To remove external lighting effects, the vessel was finished by painting the outside surface to make it opaque.

The LPE process contains a heterogeneous mixture of solvent, precursor particles and exfoliated materials. Indirect measurements of changes in concentration are obtained through an initial correlation of extinction with *ex situ* measurements of product concentration. This is performed at a suitable wavelength where changes in nanosheet morphology (thickness and length) have a negligible influence on the extinction coefficient [23]. This in-line approach can be integrated in any blender system with a few important considerations:

• Material sensitivity: This transmission spectroscopy method using an external fibre-optic probe is applicable for materials where the extinction coefficient (ɛ) is dominated by absorbance (α), where ɛ(λ)=α(λ)+ρ(λ). Graphene is one example, however, other 2DMs (e.g., h-BN) can have large scattering contributions (ρ).
• Process dynamics: During impeller rotation, there is a significant amount of signal noise created by light scattering off the bubbles and large solid particles in the system (Fig. 8 b). To address this, we developed a protocol to take measurements during a period when the equipment is off so that the bubbles coalesce, large particles sediment, and any foam can drain out (for aqueous-surfactant dispersions).
• Optical characteristics of the system: The vessel must be made completely opaque so that no other light interferes with the optical measurements.

## 5 Operation instructions

### 5.1 Preparing the sample

LPE is a top-down approach to synthesis, hence an appropriate precursor must be selected to obtain the target two-dimensional material, i.e. flake graphite for graphene, bulk MoS_2_ for MoS_2_ nanosheets, bulk h-BN for h-BN nanosheets. Once a precursor has been selected, a solvent or dispersion-assisted liquid has to be chosen. For solvents, the suitability for exfoliation and stable 2DM dispersions is typically based on matching the Hansen solubility parameters with the nanomaterial desired [24]. Unfortunately, many commonly used solvents for LPE can be toxic, have high boiling points, and tend to be incompatible with the blender vessel materials (e.g., NMP). Alternative ‘green’ solvents, such as co-solvents of water/IPA and water/ethanol [25], have been shown to produce stable dispersions, have lower boiling points than traditional solvents, and are compatible with certain kitchen blender materials (this would need to be checked beforehand on a case-by-case basis).

Liquid dispersants based on aqueous-surfactant solutions (e.g., water/Sodium Cholate (NaC)) or macromolecules can produce high concentration 2DM dispersions through either electrostatic or steric repulsion mechanisms. These solutions have good compatibility with most, if not all kitchen blenders. One disadvantage is the high shear mixing process leads to aeration and the formation of stable foams which increase the temperature and pressure inside the vessel. On the application side, surfactants can also be difficult to wash off the nanosheets, and can persist after evaporating the excess liquid, altering the material properties.

A good choice of solvent or dispersion-assisted liquid can have a drastic impact on the concentration of nanomaterial produced. The list of potential liquid dispersants has grown considerably over the last decade, with many examples in the scientific literature covering a variety of different mediums, including off-the-shelf items from single malt whiskey [26] to black tea [27].

Quantities are more nuanced, though most have found that the concentration of nanomaterial (C) produced to scale linearly with the concentration of bulk precursor, $C_{i}$ [5]. This relationship ($C\propto C_{i}$) breaks down at the extremes of precursor concentrations or when the liquid is sufficiently saturated [22]. Changes in surfactant concentration typically produce one maxima, though there are some outlier cases for lower purity graphite precursors [28]. Generally, only small amounts of surfactant are required for stabilising 2DMs, with anything above ∼10mM dropping off sharply, the maxima usually lying somewhere in this range [29]. Choice of surfactant here seems to have little effect, hence a green surfactant would be ideal for this purpose. Additionally, though LPE may have been demonstrated in a specific scenario, it may not work in the mixer; one example we have come across is water/urea co-solvent which has been demonstrated in cooled sonicated baths [30]. We recommend either aqueous-surfactant solutions, suitable macromolecules, or green co-solvents such as aqueous Isopropyl Alcohol (IPA) or Ethanol (depending on your vessel, see 5.3).

### 5.2 Pre-treatment

In addition to nanomaterial synthesis the blender can be used in the pre-treatment of Transition Metal Dichalcogenides (TMDs) such as MoS_2_. Often these materials require a ‘pre-exfoliation’ and washing step to remove water soluble or ionic impurities which act to destabilise dispersions, resulting in low or no, nanomaterial production. This has been demonstrated before via ultrasonication [29]. Comprehensive analysis of the effect of blender speed on the removal of these impurities has not been carried out however anecdotal evidence from our testing suggests that higher blender speeds result in more effective removal. The liquid containing impurities can then be removed by either allowing the precursor material to sediment and pipetting the liquid off the top, or by washing through a generic paper filter. Vacuum filtration, if available, would expedite the filtration process.

### 5.3 Vessel considerations

When selecting a vessel there are a number of considerations that should be made:

1. Is the solvent compatible with the vessel material (see Section 5.1).
2. Ensure the vessel does not exceed temperatures >60°C to avoid reliability issues, i.e. by active cooling. This is particularly important for foaming solutions (aqueous-surfactants) that increase pressure build-up inside the vessel.
3. The blender creates vibrations and acoustic noise when operating, so the location must be suitable.
4. No foreign objects are around the blender during operation.
5. All avenues of liquid release are checked and properly inhibited.

**Fig. 9:**
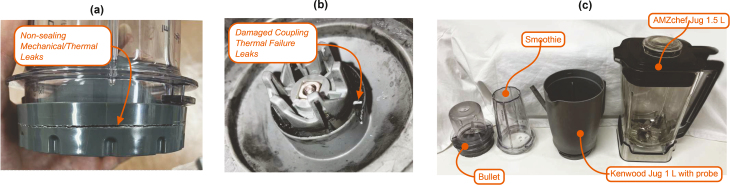
Failures of sealed vessels in (a) and (b), most occur at weak points in the vessels construction, likely due to thermal cycling weakening the material, and high pressure and temperature causing material failure. (c) Shows a labelled selection of vessels we have tested with, leakage around the lower seal was common with the Kenwood vessels, but we found this could be mitigated by increasing the pretension on the lower seal (initially achieved this with ptfe tape, then moved on to a small 3D printed shim).

In a sealed vessel, fluid temperature and subsequently vessel pressure will increase which may cause failure of the vessel material, or any sealing surfaces, resulting in leaks or catastrophic equipment failure. Thermal cycling through extended operation of this style of vessel may also weaken the materials, which combined with the high pressure could increase the risk of failure (Fig. 9); hence the recommendation in Point 2.. If the solvent compatibility is unknown, a simple patch test on a region of the vessel can be carried out. Alternatively, if feasible, a small amount of the material could be cut from the vessel housing and left in the chosen solvent overnight. A visual check is then performed to determine whether the material has been dissolved or damaged by the solvent.^4^

### 5.4 Blender operation

The blender must be operated in intervals to prevent excessive heating of the motor and the liquid dispersion in the absence of a cooling jacket. Manufacturers often recommended running for a minute *on* per minute *off*, though there is less of an issue with the more powerful blenders such as the AMZChef, where couplings and motor are of higher quality, these can be run at moderate→high loads without the risk of immediate failure. Example Arduino sketches for providing automated control are available on our OSF page. Some solvent choices may increase the risk of coupling failure, for example when the blender is operated using surfactant solutions, aeration creates a foam which increases the fluid resistance, thereby putting more strain on the blender components. Organic solvents, such as ethanol, do not suffer from this issue. Intensifying 2DM production for a particular solvent or liquid dispersant predominantly depends on increasing the process time t, rotor speed ω, impeller diameter $d_{i}$ and/or reducing the volume V [5], [6], [18]. The precise scaling of these factors is approximately in line with the energy inputted (E) to the mixture per unit volume of liquid, irrespective of the method of liquid exfoliation [5]. Power (E=Pt) for stirred batch reactors is well known to scale as follows [31]:

(1) $$P∼\rho \;\omega^{3}\;d_{i}^{5}$$

**Fig. 10:**
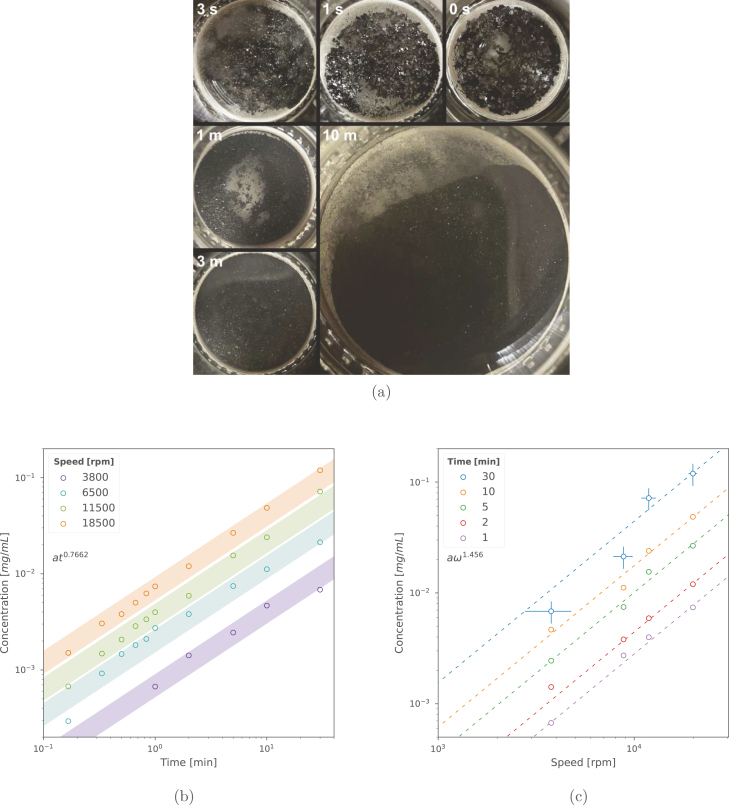
Breakdown of graphite particles occurs quickly (a). Graphene concentration is plotted against time in (b) within a window of uncertainty ($1.2\sigma_{max}$), and against speed in (c) with speed (±1000rpm) and concentration error bars ($\sigma_{max}$). We find production exponents of $t^{0.77}$ and $\omega^{1.46}$ for $C_{s}=4.3\;\text{mg/mL}$ of Sodium Cholate (NaC) at a process volume of 200mL and an average layer cutoff of 〈N〉=5 (centrifuged for 45 min at 380Relative Centrifugal Force (RCF), initial graphite concentration of $C_{i}=20\;\text{mg/mL}$). Optical extinction measurements were used to determine concentration (through the Lambert–Beer law) using a PerkinElmer Lambda 365 UV–vis Spectrophotometer, and a measured extinction coefficient of 1521 L g^−1^ m^−1^ at λ=660nm. The procedure for measuring the extinction coefficient is described in detail elsewhere [28].

Eq. (1) suggests that increasing the rotor diameter and rotational speed are the most important factors for increasing production. Through our extensive testing, we have collated a sizeable amount of data on running the Kenwood blender with an optimum amount of NaC ($C_{s}=4.3\text{g}/\text{L}$). This is presented in Fig. 10, Fig. 10. Note too, that at shear rates under a critical value (${\overset{˙}{\gamma }}_{c}\approx 1000\;{\text{s}}^{-1}$ for graphene), the mechanical forces inside the blender vessel are too low to overcome the interstitial van der Waals forces of the layered precursor [5]. Another confounding issue is the instability in rotor speed at low power values, where the variance in rotor speed (usually ±1000rpm) can reach as high as ±2000 (for open-loop blenders) as the rotor repeatedly stalls and picks up speed. For these reasons, we recommend running at speeds above ≈4000rpm.

The highest yields were observed when using the smaller, bullet-style vessel at moderate speeds (≈10krpm), where most of the power is dissipated into the fluid (2% mass yield of 5 layer average graphene after 30 min *on*, using the Kenwood with a water/NaC dispersant). This yield and production rate is favourable compared to existing and more expensive laboratory methods (e.g., ultrasonication). Separation and characterisation of the material after processing is explained in more detail within Section 6.

LPE produces nanosheets with a range of lengths typically 10nm to 1000nm, however, nanosheet length can depend on the solvent chosen. Varrla et al. showed with surfactant mixtures, that the largest, thinnest nanosheets are produced the Critical Miscelle Concentration (CMC) [6]. They also showed that increasing the rotor speed does slightly increase the length of sheets produced, with other factors having a negligible effect. More recently, surfactant concentrations of ≈10mM were shown to be a critical point for concentration, length and thickness, irrespective of the surfactant choice or CMC [29]. LPE tends to produce nanosheets within large size distributions, though it is possible to reprocess any separated supernatant to reduce the average layer count [18], [28].

### 5.5 Recycling

Once processing has completed, and all nanomaterial has been removed from the batch, the precursor sediment can be recycled using a fresh batch of solvent/liquid dispersant. We have found that recycling this sediment using a second exfoliation run and fresh aqueous-surfactant solution can produce ≈70% of the initial 2DM output [28]. One can also try exfoliation in recovered solvents, or upcycling other layered precursor sources as Stafford and Kendrick showed in their recent paper on end-of-life Li-ion battery anode materials [28].

### 5.6 Post processing

Whilst post processing is not specifically related to the hardware in discussion, in this paper it is nonetheless an important step in the synthesis of nanomaterial dispersions. The most common method for post processing is to centrifuge liquid exfoliated dispersions to sediment out large, unexfoliated material. There are also other methods available to separate out size fractions such as Liquid Cascade Centrifugation (LCC) [32] in which nanosheets of specific sizes can be isolated using multiple centrifugation steps. It is also possible to obtain powders of the synthesised 2DM dispersion by evaporating the liquid after separating the 2DM from the bulk precursor. When using surfactant-assisted methods, the surfactant adheres to the 2DM making it difficult to wash off completely. Co-solvents such as ethanol or IPA are better suited for synthesising powders for this reason, as they boil off at low temperatures, leaving the remaining nanomaterial. Using the dispersions directly from the LPE process remains the easiest way to implement the 2DM in applications. However, this depends on the application and the mass fraction of few-layer nanosheets in dispersion that are required to obtain suitable properties.

## 6 Validation and characterisation

### 6.1 UV-vis-NIR spectroscopy

**Fig. 11:**
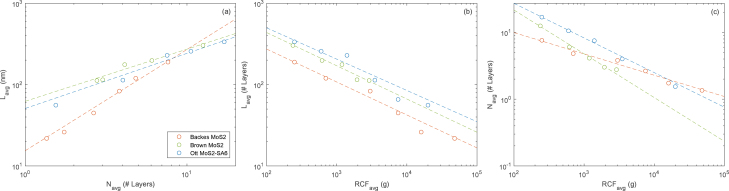
Graphs of average nanosheet layer number ($N_{\mathrm{avg}}$), and average nanosheet length ($L_{\mathrm{avg}}$), against (a) each other, (b,c) average Relative Centrifugal Force (RCF) value in Liquid Cascade Centrifugation (LCC) post processing. Data collated from Backes [34], Ott [33], and the authors of this work (Brown MoS2). All data has been plotted on log–log axes and power law fits are to guide the eye, as in Backes et al. original plots.

The hardware described has been used effectively to synthesise few-layer graphene, h-BN and MoS_2_ dispersions in both surfactant solutions (aqueous-sodium cholate), and organic co-solvents (aqueous-ethanol). These can be easily isolated from residual bulk material by centrifugation. Fig. 11 shows that MoS_2_ dispersion characteristics (mean nanosheet length, mean nanosheet layer number) are comparable to those reported in literature using tip sonication [33], [34]. Data for other materials (Graphene, WS_2_, MoS_2_, and more), and methods (bath sonication) show similar trends [33], [34]. MoS_2_ dispersion characteristics from the authors of this work (‘Brown MoS_2_’ in Fig. 11) are evaluated using optical-spectroscopy metrics [35].

**Fig. 12:**
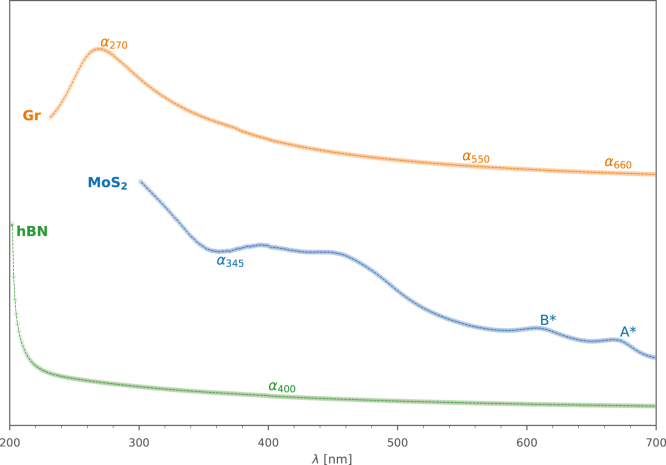
Three different UV–vis extinction spectra of materials (Y is offset, arbitrary units) created with our mixer, graphene (Gr), MoS_2_ and h-BN, along with the salient points for establishing the thickness, concentration and/or length [23], [35], [36].

Optical extinction can be easily measured at any point along the UV–vis–NIR spectra, but the value at certain points along the response curve is affected by the morphology of the nanosheets. For instance, the spectra for Gr shows a distinct absorbance peak near 270nm (Fig. 12), indicative of the electronic conjugation of graphene. The height of this peak relative to the absorbance at longer visible wavelengths has been shown to increase with decreasing layer numbers [23]. In the longer wavelength region (600–800nm), extinction is not affected by nanosheet morphology rather acting solely in relation to the concentration of nanomaterial within the sample. If the material in question is functionalised, these changes are visible in the characteristic UV–vis–NIR response (e.g. the absorbance peak for graphene oxide is at 230nm unlike that of pristine graphene). Therefore, UV–vis–NIR spectroscopy allows for an inexpensive and rapid indication of the morphology of the nanosheets.

### 6.2 Microscopy

**Fig. 13:**
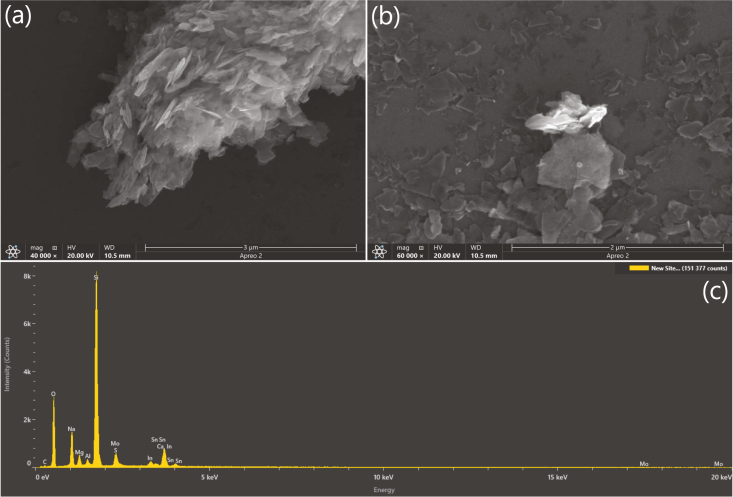
(a, b) Scanning Electron Microscopy (SEM) images of exfoliated MoS_2_ deposited on Indium Tin Oxide (ITO) glass slide. (a) shows a clumped deposition of flakes, (b) shows a more uniform distribution of flakes deposited on the surface. (c) shows an Energy Dispersive X-ray Spectroscopy (EDS) spectra of the deposit with a clear peak in the 2–2.5 keV range indicating the presence of Mo (2.293 keV) and S (2.307 keV).

**Fig. 14:**
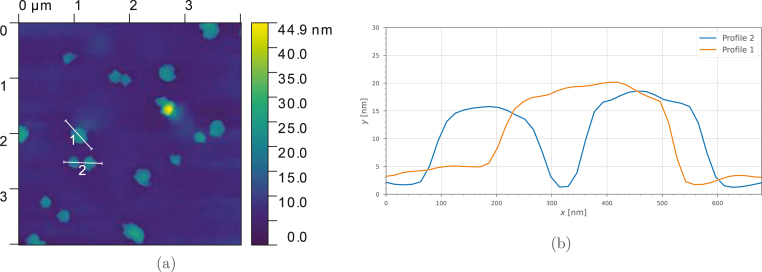
Atomic Force Microscopy (AFM) of graphene nanoplatelets 〈L〉≈200nm washed and then exfoliated in IPA and water (50:50 mix by mass) note that Sodium Cholate (NaC) can obscure Atomic Force Microscopy (AFM) as it adheres to the surface of the nanoplatelets causing the probe tip to stick and drag. The exfoliated nanosheets have an apparent thickness of around 13nm. For this, 10μL of solution was drop cast onto a freshly cleaved and heated mica sheet ($0.5\times 0.5\;{\text{cm}}^{2}$), as described by Backes et al. [37].

As well as optical spectra, MoS_2_ synthesis has been validated by observing deposits under Scanning Electron Microscopy (SEM) and taking Energy Dispersive X-ray Spectroscopy (EDS) measurements, as seen in Fig. 13. Graphene has been validated using Atomic Force Microscopy (AFM) (see Fig. 14), where we find good agreement with existing literature [6], [21], and with spectroscopic layer number estimations 〈N〉 defined by Backes et al. [23]. Single layer height for graphene has generally been found to be between 1 and 2nm measured with AFM which is much larger than the theoretical value [37]. We have found that surfactants obscure AFM measurements due to their adherence onto the surface of the FLG sheets. Removing them is difficult, but can be achieved through a combination of dilution and washing the sample once prepared [37]. Though graphite does not typically require a pre-washing step (described in Section 5.2) as opposed to TMDs where this step is a prerequisite, for AFM measurements this was introduced as a further cleaning step to obtain a reliably good image.

## 7 Conclusion

**Fig. 15:**
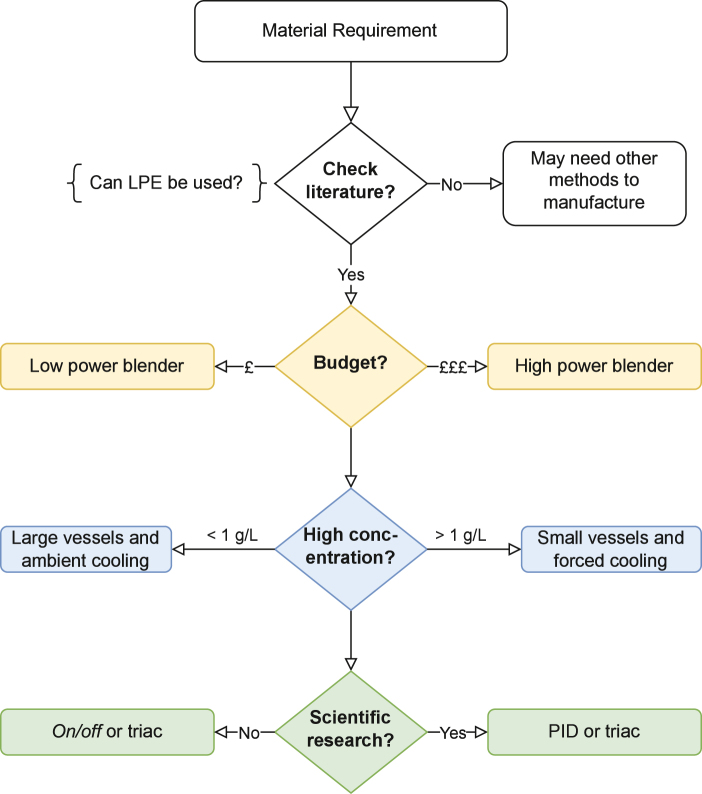
Simplified decision tree explaining the various aspects involved in deciding on a Liquid Phase Exfoliation (LPE) system.

We have designed a low-cost, open-source liquid-phase exfoliation process for producing atomically thin materials and demonstrated the synthesis of few-layer graphene, MoS_2_ and h-BN nanosheets. OpenLPE is a mechanochemical process based off traditional household kitchen blenders that have been adapted to provide precise control over key shear-exfoliation parameters that influence nanosheet concentration and morphology. Automated control was developed and implemented to ensure repeatable synthesis conditions (fluid shear stresses, batch temperature) and hardware reliability. Detailed descriptions of the hardware and software for controlling impeller speed, synthesis duration, process temperatures and measuring inline spectroscopic characteristics have been provided. These have been outlined in a modular framework, with the aim of allowing researchers to decide which components to choose for their application. Each module has an accompanying repository containing codes, designs and build instructions to facilitate this. To simplify the selection process, a short diagram is provided in Fig. 15, explaining the decisions which are useful for establishing what mixer and adaptations the user may need.

Validation of the hardware for the synthesis of 2D materials was used to identify process restrictions and performances. The range of solvents that are compatible with blender vessels can be a limitation. However, this can also indirectly promote green synthesis strategies. The need to use aqueous-surfactant or other mild co-solvent methods inherently avoids the use of toxic solvents which have high boiling points (e.g. NMP). Glass vessels are available and would broaden the range of solvents if required. In this arrangement, an examination of the compatibility of the seals around the impeller would be necessary to prevent leaks.

The investigations on graphene synthesis revealed a minimum 20% increase in concentration when moving from jug to bullet type vessels. These smaller vessels were found to be advantageous for low volume batches (<160 mL), achieving 1.4%wt yield in 15 min compared to ≈0.5%wt for the larger jug type. Active cooling is required to operate the small vessel process reliably and we demonstrated this without using expensive ancillary heat pumps. Using MoS_2_ as a model 2D material, we showed that the quality of the shear-exfoliated product compares favourably to contemporary synthesis approaches like ultrasonication. Furthermore, there are other advantages that are attractive for materials processing. This liquid-phase exfoliation system is capable of processing nanosheet dispersions from dilute to ∼100 g/L concentrations. This robustness across low/high solids content and liquid viscosity make it an appropriate technique for broad laboratory research and suitable for numerous applications including functional inks, pastes and composites. Overall, this hardware solution can lower entry cost restrictions and facilitate further advancements in 2D materials and other mechanochemical research fields.

## CRediT authorship contribution statement

**Diego T. Pérez-Álvarez:** Conceptualization, Methodology, Software, Investigation, Formal analysis, Visualization, Writing – original draft. **Jacob Brown:** Investigation, Formal analysis, Visualization, Writing – original draft. **Jason Stafford:** Conceptualization, Methodology, Software, Investigation, Formal analysis, Visualization, Writing – review & editing, Supervision, Funding acquisition.

## Declaration of competing interest

None

## Footnotes

1 It also reduces human input, as the equipment is often run for several hours at a time.

2 Sometimes called a thyristor or Silicon Controlled Rectifier (SCR).

3 If printing in PLA note that this material is transparent to infra-red, so the interrupting ring should have something opaque stuck to the inside (sand-paper works)

4 Our testing has shown that the AMZchef is resistant to ethanol mixtures. The Kenwood vessels used in this work were not resistant to ethanol or Cyrene.
